# Functional elucidation of miR-494 in the tumorigenesis of nasopharyngeal carcinoma

**DOI:** 10.1007/s13277-015-3356-8

**Published:** 2015-03-26

**Authors:** Hong-Fang Duan, Xiao-Qing Li, Hong-Yi Hu, Yu-Chi Li, Zhi Cai, Xue-Shuang Mei, Peng Yu, Li-Ping Nie, Wei Zhang, Zhen-Dong Yu, Guo-Hui Nie

**Affiliations:** 1 Department of Otolaryngological, Peking University Shenzhen Hospital, 518036 Shenzhen, Guangdong Province China; 2 Guangzhou Medical University, 510000 Guangzhou, Guangdong Province China; 3 Department of Clinical Laboratory, Peking University Shenzhen Hospital, 518036 Shenzhen, Guangdong Province China; 4 Shantou University Medical College, 515041 Shantou, Guangdong Province China; 5 Guangdong and Shenzhen Key Laboratory of Male Reproductive Medicine and Genetics, Shenzhen PKU-HKUST Medical Center, Institute of Urology, Peking University Shenzhen Hospital, 518036 Shenzhen, Guangdong Province China; 6 Central Laboratory, Peking University Shenzhen Hospital, 518036 Shenzhen, Guangdong Province China; 7 Biomedical Research Institute, Shenzhen Peking University—the Hong Kong University of Science and Technology Medical Center, 518036 Shenzhen, Guangdong Province China

**Keywords:** Nasopharyngeal carcinoma, miR-494, Tumor suppressor, GALNT7, CDK16

## Abstract

Nasopharyngeal carcinoma has very high incidence and high mortality worldwide. MiRNA is related to the tumorigenesis and metastasis of a variety of tumors. In the present study, we verify that the expression of miR-494 in NPC tissues and NPC-derived cells was down-regulated, respectively. The proliferation, colony formation, migration, and invasion of NPC-derived cells were suppressed, while the cell apoptosis was promoted, when miR-494 was over-expressed in these cells. GALNT7 and CDK16 were confirmed to be the direct targets of miR-494. These results suggested that miR-494 play an inhibitory role in the tumorigenesis of NPC.

## Introduction

Nasopharyngeal carcinoma (NPC) is a non-lymphomatous, squamous cell malignancy arising from the epithelial cells of the nasopharynx [1]. Some epidemiological reports about NPC revealed its extremely unbalanced endemic distribution, with the highest morbidity in South China [2]. The standard treatment is radiotherapy for early-stage NPC and chemoradiotherapy for advanced NPC around the world at the present time [3]. Since NPC is highly sensitive to radiation, the most common and effective treatment to NPC patients is primarily based on radiotherapy. Besides, concurrent adjuvant chemotherapy can also increase survival rates [4]. However, early diagnosis is difficult due to the location of the tumor and the lack of clinical manifestation. In addition, NPC is highly invasive and metastatic. Once metastasis occurs, the prognosis of patients is poor [5–7]. Thus, it is crucial to investigate the underlying mechanism for NPC tumorigenesis and metastasis.

MicroRNAs (miRNAs) are endogenous noncoding small single chain RNAs composed of ∼22 nucleotides which target the 3′ untranslated regions (3′-UTRs) of certain genes to regulate their expression [8–10]. MiRNAs have broad effect on tumorigenesis and tumor biological processes including tumor development, differentiation, metastasis, and chemoresistance [11]. They may act as tumor suppressors or oncogenes to play critical roles in carcinogenesis [12]. Recent studies have reported that abnormal expression of miRNAs involved in NPC development and progression by regulating cell growth, proliferation, apoptosis, invasion, and metastasis [13–16], indicating that miRNAs play important roles in NPC tumorigenesis. MiR-494 is located on chromosome 14q32.31 [17]. Previous studies revealed ectopic expression of miR-494 in liver cancer, lung cancer, gastrointestinal cancer, brain tumor, and so on [18–21]. However, the potential role of miR-494 in NPC progression has still not been clarified. In the present study, we examined the expression of miR-494 in NPC specimens and NPC-derived cells, and investigated the role of miR-494 in NPC-derived cell lines by cell proliferation, colony formation, migration, invasion, and apoptosis assays. We also identified polypeptide N-acetylgalactosaminyltransferase 7 (GALNT7) and cyclin-dependent kinase 16 (CDK16) as potential targets of miR-494. Overall, we provided evidence showing that miR-494 may play an inhibitory role in the carcinogenesis of NPC.

## Materials and methods

### Specimens

A total of 20 fresh nasopharyngeal carcinoma specimens and 20 fresh normal nasopharyngeal tissues were obtained from the Peking University Shenzhen Hospital and Shenzhen People’s Hospital (Shenzhen, Guangdong, China). All the NPC samples were diagnosed as non-keratinizing squamous cell nasopharyngeal carcinoma without chemotherapy or radiotherapy before the surgery, and the fresh nasopharyngeal tissues from healthy individuals were diagnosed without any other medical illness. All specimens were frozen in liquid nitrogen for further study. The clinicopathological information of the patients is shown in Table 1. Staging was performed by the 2003 UICC staging system. All patients were informed of the purposes of this research and signed the written consent.

**Table 1:** Clinical and pathological characteristics in NPC patients

Characteristics	Number of cases
Mean age range (years)	44 (28–61)
Sexual distinction	
Male/female	16/4
Degree of differentiation	
Undifferentiated/differentiated	14/6
Histology	
Squamous/others	20/0
Lymph node metastasis	
Positive/negative	16/4
Distal metastasis	
Positive/negative	0/20
Clinical TNM stage	
(I–II/III–IV)	3/17

### Cell culture

Human nasopharyngeal epithelial cell line (NP69) and three NPC cell lines (6-10B, 9-4E, CNE2) were used in this study. The 6-10B and CNE2 cells were generous gifts from Southern Medical University (Guangzhou, Guangdong, China). The other two cells, 9-4E and NP69, were provided by Peking University Shenzhen Hospital. All NPC cell lines were cultured in RPMI 1640 (Gibco, Carlsbad, CA, USA), with 10 % fetal bovine serum (Gibco, Carlsbad, CA, USA), 1 % antibiotics (100 μ/ml penicillin and 100 mg/ml streptomycin sulfates), and 1 % glutamate (Gibco, Carlsbad, CA, USA), and then incubated at 37 °C in a humidified chamber containing 5 % CO_2_.

### Extraction of total RNA, reverse transcription, and quantitative real-time polymerase chain reaction

TRIzol reagent (Invitrogen, Carlsbad, CA, USA) was used to extract the total RNA from clinical specimen tissues according to the user manual. 6-10B, 9-4E, or CNE-2 (4 × 10^5^ cells/well) was plated into six-well plates (BD Biosciences, USA) with three replicate wells, respectively. The cells were trypsinized to extract the total RNA by miRneasy Mini Kit (Qiagen, Valencia, CA, USA) 24 h later. The RNA samples with 260/280 ratios of 1.8–2.0 were used for further experiments. Total RNA was converted into cDNA by using the miScript II RT Kit (Qiagen, Valencia, CA, USA) or PrimeScript^TM^ RT reagent Kit (TaKaRa, Japan). The expression level of miR-494 was confirmed with the miScriptSYBR®green PCR Kit (Qiagen, Valencia, CA, USA), and the mRNA expression levels of GALNT7 and CDK16 were confirmed with the SYBR® Premix Ex Taq^TM^ II (TaKaRa, Japan), respectively, according to the manufacturer’s instructions on the Roche lightcycler 480 Real-Time PCR System. U6 and β-actin were used as the endogenous control to normalize the data. Their expression levels were shown as fold differences relative to the U6 and β-actin, which was based on the equation relative quantification (RQ) = 2^−ΔΔCt^ [ΔΔCt = (meanCt_cancer_ − meanCt_control_) − (meanCt_normal_ − meanCt_control_)] [15]. Moreover, the miR-494 forward primer was 5′-UGA AAC AUA CAC GGG AAA CCU C-3′ and the U6 forward primer was 5′-ACG CAA ATT CGT GAA GCG TT-3′, and their reverse primer was the universal primer supported by the miScriptSYBR®green PCR Kit (Qiagen, Valencia, CA, USA). The GALNT7 mRNA forward primer was 5′-GGG ATT ATT TGC CAT TGA ACG A-3′ and the reverse primer was 5′-AGA CGG TAG ATA TGT CCA ACA C-3′, the CDK16 mRNA forward primer was 5′-GCA CGA GGA CTT GAA GAT GG-3′ and the reverse primer was 5′-CGC ATA CGC ACT CTC ACT G-3′, the β-actin primer forward primer was 5′-GGC ACC ACA CCT TCT ACA ATG AG-3′ and the reverse primer was 5′-GGA TAG CAC AGC CTG GAT AGC A -3′.

### Cell transfection

All cells were transfected with miR-494 mimic(5′-UGA AAC AUA CAC GGG AAA CCU C-3′), negative control(5′-UUC UCC GAA CGU GUC ACG UTT-3′), inhibitor(5′-GAG GUU UCC CGU GUA UGU UUC A-3′), or inhibitor negative control(5′-CAG UAC UUU UGU GUA GUA CAA-3′) (GenePharma, Shanghai, China) using the Lipofectamine 2000 (Invitrogen, Carlsbad, CA, USA), which were mixed in the Opti-MEM® I Reduced Serum Medium (Gibco, Carlsbad, CA, USA) after plating 24 h. Then, fluorescence microscopy and the quantitative real-time polymerase chain reaction (qRT-PCR) were used to verify transfection efficiency.

### Cell proliferation assay and colony formation assay

The 3-(4,5-dimethylthiazol-2-yl)-2,5-diphenyltetrazoliumbromide assay (MTT, 5 mg/ml, Sigma-Aldrich) was used to analyze the cell proliferation. 6-10B, 9-4E, and CNE2 (6,000 cells/well) with five replicate wells of each condition were plated into 96-well plates. The blank control wells were just set up with medium. Cell proliferation was assessed at 0, 24, 48, or 72 h post-transfection. Before the meterage, cells were stained with 20 μl MTT and incubated at 37 °C in a humidified chamber containing 5 % CO_2_ for 4 h. Then, the MTT medium mixtures were discarded, and 150 μl dimethyl sulfoxide (DMSO, Sigma, Shanghai, China) was added. After shaking for 10 min at room temperature, the absorbance was measured by the ELISA microplate reader (Bio-Rad, Hercules, CA, USA) at a wavelength of 490 nm (with 630 nm as the reference wavelength).

A total of 1,000 cells from each group 24 h after transfection were cultured in the six-well plate for 10 days. Then, cells were washed with phosphate-buffered saline (PBS). Cells were fixed with paraformaldehyde for 25 min, stained with 0.1 % crystal violet (Sigma-Aldrich) for 25 min, and then washed three times. The colonies were recorded and counted. All phases were performed in triplicate.

### Cell migration and invasion assay

For transwell migration assays, 1 × 10^4^ 6-10B, 9-4E, or CNE-2 cells were harvested 24 h post-transfection. Then they were plated into the upper chambers (24-well insert, pore size 8 μm, Corning) with 100 μl serum-free 1640 medium. The lower chambers were filled with 500 μl RPMI 1640 containing 10 % fetal bovine serum. Then cells were cultured at 37 °C in a humidified chamber containing 5 % CO_2_. Two days later, the cells on the surface of the upper chamber were swapped with cotton gently. Cells under the surface of the lower chamber were washed with PBS, fixed with paraformaldehyde for 25 min, stained with 0.1 % crystal violet for 25 min, and then washed three times.

For invasion assays, Matrigel (1:5, 50 μl/well, BD Bioscience, San Jose, CA, USA) was added to transwell chambers in 24-well plate. The next steps were the same with the migration assays. Finally, migrated and invaded cells were counted by taking photographs in three fields. All experiments were performed in triplicate.

### Cell scratch assay

Cell scratch assay was also used to examine the migration of NPC cells. Around 3 × 10^5^ 6-10B, 9-4E, or CNE-2 cells were seeded on 12-well plates 1 day before the transfection. The cells were transfected when they grew to reach almost total confluence, and a sterile 200-μl pipette tip was used to scrape a clear line through the cell layer 6 h later. Then, the medium was changed with serum-free 1640 medium. Migration distance was observed under the inverted microscope and imaged at the time of 0, 12, and 24 h. Experiments were performed in triplicate.

### Cell apoptosis assay

For cell apoptosis analysis, 2 × 10^5^ 6-10B, 9-4E, or CNE-2 cells were seeded on six-well plates. The cells were incubated at 37 °C in a humidified chamber containing 5 % CO_2_ for 48 h after transfection. The cells were collected and washed twice with pre-chilled PBS. Then they were resuspended in 1× binding buffer and stained with Annexin V-FITC (AV, 5 μl) and propidium iodide (PI, 3 μl) by using the annexin V-fluorescein isothiocyanate (FITC)/PI detection kit (Invitrogen, Carlsbad, CA, USA). Finally, flow cytometry (EPICS, Xl-4, Beckman, CA, USA) was used to quantify the percentage of apoptotic cells after mixed for 15 min at room temperature. Each experiment was done at least three times.

### Luciferase reporter assay

The miRNAs target sequences were inserted between the XhoI–NotI restriction sites in the 3′-UTR of the hRluc gene of the psiCHECK^TM^-2 luciferase vector (Promega, Madison, WI, USA) to generate reporter constructs. The primer sequences for the 3′-UTR of GALNT7 mRNA (forward primer 5′-CCG CTC GAG ATT GTC CAC TGA CAT TTG GGA TTT A-3′ and reverse primer 5′-AAG GAA AAA AGC GGC CGC CAA ACT TCC TCT GGC AGT AGT TTG T-3′) and CDK16 mRNA (forward primer 5′-CCG CTC GAG TCA TAC CAG CCC CCA GGA CCA CTA C-3′ and reverse primer 5′-AAG GAA AAA AGC GGC CGC GTT CCA AAT AGG GGC TGT GTC CCT G-3′) were designed. They contain the potential binding sites to verify the binding sites of the miR-494. Meanwhile, G and T, and A and C were substituted to mutate the potential binding sites (Fig. 6a, c). All these four short fragments were cloned into psiCHECK^TM^-2 luciferase vectors, respectively, and all the constructs were verified by sequencing. They were transfected together with miR-494 mimic, negative control, inhibitor, or inhibitor negative control into 6-10B, 9-4E, or CNE2 cells in three replicate wells. After 48 h, the dual luciferase assay system (Promega, Madison, WI, USA) was used to detect the luciferase activity according to the manufacturer’s instructions. Normalized data were analyzed by the quotient of Renilla/firefly luciferase activities. The experiments were repeated at least three times.

### Western blotting

A total of 3 × 10^5^ 6-10B, 9-4E, or CNE-2 cells were seeded on six-well plates and then transfected as mentioned above. Radio immunoprecipitation assay (RIPA) lysis buffer (Sigma, USA) was used to lyse the cells 48 h later. A total of 20 μg protein sample was loaded in each well and separated with 10 % sodium dodecyl sulfate polyacrylamide gel electrophoresis (SDS-PAGE). The proteins were then electroblotted onto polyvinylidene difluoride (PVDF) membrane (Millipore, Billerica, MA, USA) with wet blotting. After being blocked in blocking buffer (1× Tris-buffered saline (TBS), 0.1 % Tween-20, and 5 % nonfat milk) for 2 h at room temperature, the membranes were incubated with rabbit anti-GALNT7 (1:2,000, Abcam, USA), or anti-CDK16 (1:2,000, Proteintech, China), or anti-β-actin (1:1,000, Santa, USA) overnight at 4 °C, followed by incubation with an horseradish peroxidase (HRP)-linked secondary antibody (1:10,000, EarthOx, LLC, USA) for 1 h at room temperature. At last, the Chemiluminescence Phototube-HRP kit (WBKLS0500, Millipore) was used to visualize the immunoreactive bands.

### Statistical analysis

All data were presented as the mean ± standard deviation (*x* ± SD) from the three independent experiments. All data were analyzed by the SPSS 19.0 statistical software (SPSS Inc. Chicago, IL, USA). MTT data were analyzed by analysis of variance (ANOVA). The clinicopathological information of the patients was analyzed by chi-square (*χ* ^2^) test, while other data were determined by Student’s *t* test. Furthermore, *p* < 0.05 was considered to be statistically significant.

## Results

### MiR-494 expression is down-regulated in human NPC clinical specimens and NPC-derived cells

**Fig. 1:**
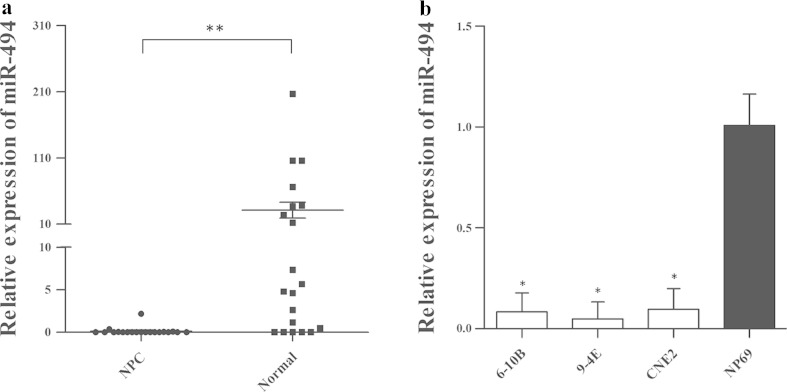
The expression of miR-494 in NPC tissues and NPC-derived cells. **a** MiR-494 is down-regulated in NPC qRT-PCR of miR-494 expression relative to U6 expression in 20 NPC tumor samples compared to 20 normal nasopharyngeal epithelial tissues. **b** qRT-PCR showed that miR-494 was down-regulated in 6-10B, 9-4E, and CNE2 cells compared with NP69 cell. 2^−ΔΔCT^ method was used to analyze the data, and the data are presented as mean ± SD (*p < 0.05, **p < 0.001)

The expression of miR-494 was examined in 20 freshly frozen NPC clinical specimens and 20 normal nasopharyngeal epithelial tissues. As shown in Fig. 1a, the expression of miR-494 was significantly down-regulated in NPC tissues compared with that in normal nasopharyngeal epithelial tissues.

We also analyzed the expression of miR-494 in three NPC cell lines (6-10B, 9-4E, and CNE2) and normal nasopharyngeal epithelial cell line (NP69). As shown in Fig. 1b, miR-494 expression was lower in 6-10B, 9-4E, and CNE2 cells (*p* = 0.001, *p* = 0.001, *p* = 0.001) than that in NP69 cells, which is in accordance with the expression pattern of miR-494 in clinical tissues.

### Validation of cell transfection efficiency

**Fig. 2:**
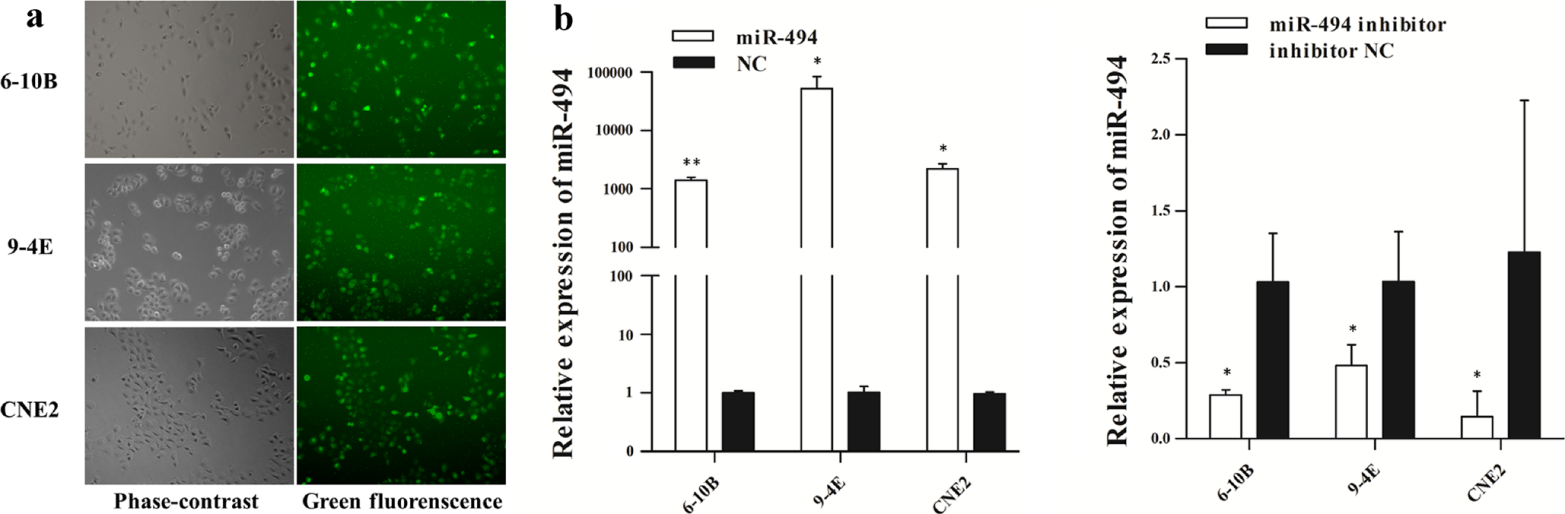
Validation of cell transfection efficiency. **a** Phase-contrast and green fluorescence images were taken from the same field. **b** The relative expression levels of miR-494 in 6-10B, 9-4E, and CNE2 cells transfected with miR-494 mimic, negative control, miR-494 inhibitor, or inhibitor negative control. Data are presented as mean ± SD (*p < 0.05, **p < 0.001)

As shown in Fig. 2a, the transfection efficiency was more than 90 % when the cells were transfected with fluorescence-conjugated miRNA. qRT-PCR was used to verify the transfection effect and revealed that miR-494 was over-expressed obviously after transfection with miR-494 mimic in 6-10B (*p* < 0.001), 9-4E (*p* = 0.045), and CNE2 (*p* = 0.001) cells, and decreased after transfection with miR-494 inhibitor in 6-10B (*p* = 0.019), 9-4E (*p* = 0.005), and CNE2 (*p* = 0.010) cells (Fig. 2b).

### MiR-494 restrains the NPC cells proliferation and colony formation

**Fig. 3:**
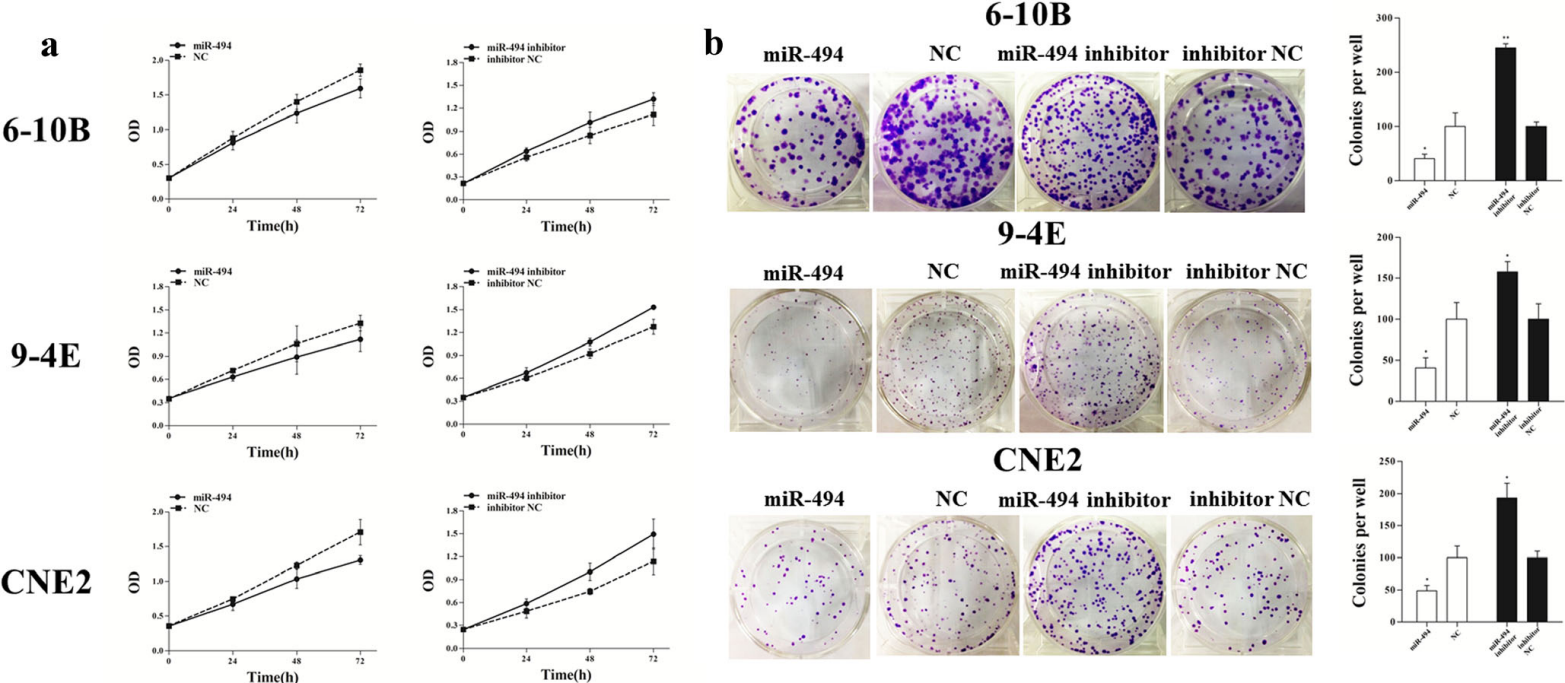
MiR-494 restrains the NPC cells proliferation and colony formation. **a** Cell growth was measured by MTT assay at different time intervals. **b** Representative images of colony formation assay of miR-494-transfected 6-10B, 9-4E, and CNE2 cells. Data are presented as mean ± SD (*p < 0.05, **p < 0.001)

MTT assay and colony formation assay were performed to observe whether differential expression of miR-494 affected the proliferation ability of NPC cells. MTT assay showed that the cell proliferation was significantly decreased by 7.9 % (24 h), 15.9 % (48 h), and 25.0 % (72 h) in 6-10B cells (*p* < 0.001); 11.8 % (24 h), 16.2 % (48 h), and 15.9 % (72 h) in 9-4E cells (*p* = 0.048); and 10.1 % (24 h), 15.7 % (48 h), and 23.5 % (72 h) in CNE2 cells (*p* < 0.001), respectively, with miR-494 mimic transfection compared with that with negative control transfection. Furthermore, the cells transfected with miR-494 inhibitor revealed obvious promotion of cell growth compared with those transfected with inhibitor negative control. The promotion rates of cell proliferation were 14.3 % (24 h), 20.0 % (48 h), and 18.0 % (72 h) in 6-10B cells (*p* = 0.006); 11.4 % (24 h), 16.4 % (48 h), and 20.1 % (72 h) in 9-4E cells (*p* < 0.001); and 20.9 % (24 h), 34.2 % (48 h), and 31.1 % (72 h) in CNE2 cells (*p* = 0.001) (Fig. 3a).

In addition, NPC cells transfected with miR-494 mimic displayed fewer and smaller colonies than those transfected with negative control, and the inhibition rates of colony formation were 59.1 % (*p* = 0.017) in 6-10B cells, 59.3 % (*p* = 0.013) in 9-4E cells, and 51.1 % (*p* = 0.012) in CNE2 cells. On the other hand, the cells transfected with miR-494 inhibitor showed more and larger colonies than those transfected with inhibitor negative control, and the promotion rates of colony formation significantly increased by 145.4 % (*p* < 0.001), 57.6 % (*p* = 0.012), and 93.3 % (*p* = 0.003) in 6-10B, 9-4E, and CNE2 cells, respectively (Fig. 3b).

### MiR-494 suppresses NPC cells migration and invasion

**Fig. 4:**
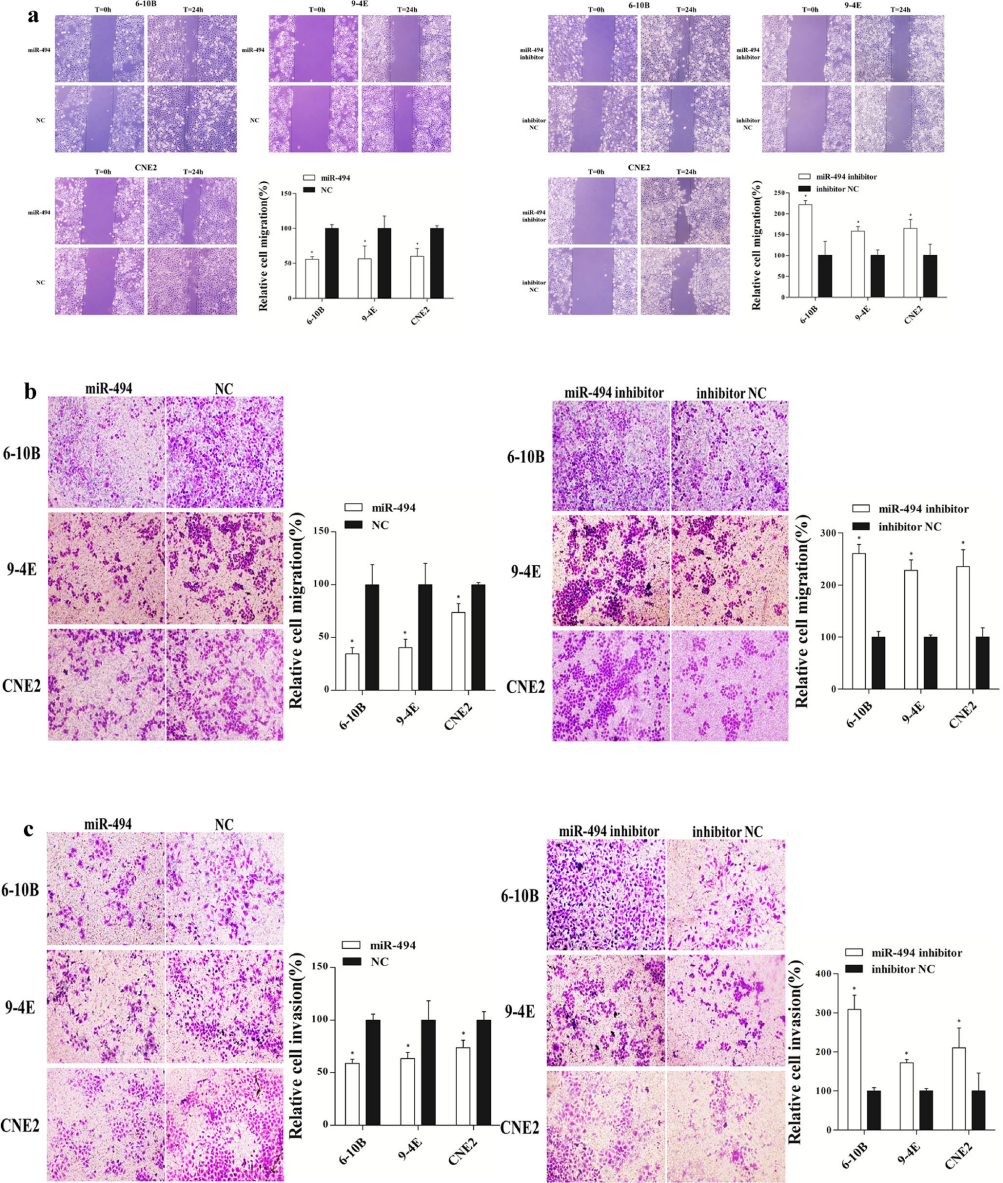
MiR-494 suppresses NPC cells migration and invasion. **a**, **b** Transwell migration assays and cell scratch assay were used to examine the 6-10B, 9-4E, and CNE2 cells’ migration after transfection with miR-494 mimic, negative control, miR-494 inhibitor, or inhibitor negative control. **c** Transwell invasion assays showed the cells’ invasion after transfection with miR-494 mimic, negative control, miR-494 inhibitor, or inhibitor negative control. Data are presented as mean ± SD (*p < 0.05, **p < 0.001)

The cell migration was examined by transwell migration assay and cell scratch assay. Transwell migration assay showed that the migration ability of cells transfected with miR-494 mimic was reduced significantly compared with those transfected with negative control. The number of migrated cells was decreased by 40.5 % (*p* = 0.005) in 6-10B cells, 59.5 % (*p* = 0.009) in 9-4E cells, and 26.3 % (*p* = 0.006) in CNE2 cells. At the same time, cell migration ability was obviously increased after transfected with miR-494 inhibitor compared with that transfected inhibitor negative control. The number of migrated cells was increased by 145.9 % (*p* < 0.001) in 6-10B cells, 128.3 % (*p* < 0.001) in 9-4E cells, and 135.8 % (*p* = 0.003) in CNE2 cells (Fig. 4a).

The same effect appeared in the cell scratch assay. The inhibition rates of migration were 41.2 % for 6-10B cells (*p* < 0.001), 43.6 % for 9-4E cells (*p* = 0.042), and 39.9 % for CNE2 cells (*p* = 0.004). The migration ability, meanwhile, was stronger in cells transfected with miR-494 inhibitor than those transfected with inhibitor negative control. The increase of migration were 121.7 % for 6-10B cells (*p* = 0.004), 58.2 % for 9-4E cells (*p* = 0.004), and 64.8 % for CNE2 cells (*p* = 0.031) (Fig. 4b).

Transwell invasion assay also demonstrated that the invasion ability was extremely weaker in cells transfected with miR-494 mimic than those transfected with negative control and was also stronger in cells transfected with miR-494 inhibitor than those transfected with inhibitor negative control. The number of invaded cells was decreased by 44.3 % (*p* < 0.001) in 6-10B cells, 62.8 % (*p* = 0.029) in 9-4E cells, and 62.8 % (*p* = 0.013) in CNE2 cells after transfection with miR-494 mimic. In contrast, the number of invaded cells was increased by 209.1 % (*p* = 0.001) in 6-10B cells, 71.8 % (*p* < 0.001) in 9-4E cells, and 110.6 % (*p* = 0.049) in CNE2 cells after transfection with miR-494 inhibitor (Fig. 4c).

### Effects of miR-494 on the apoptosis in NPC cells

**Fig. 5:**
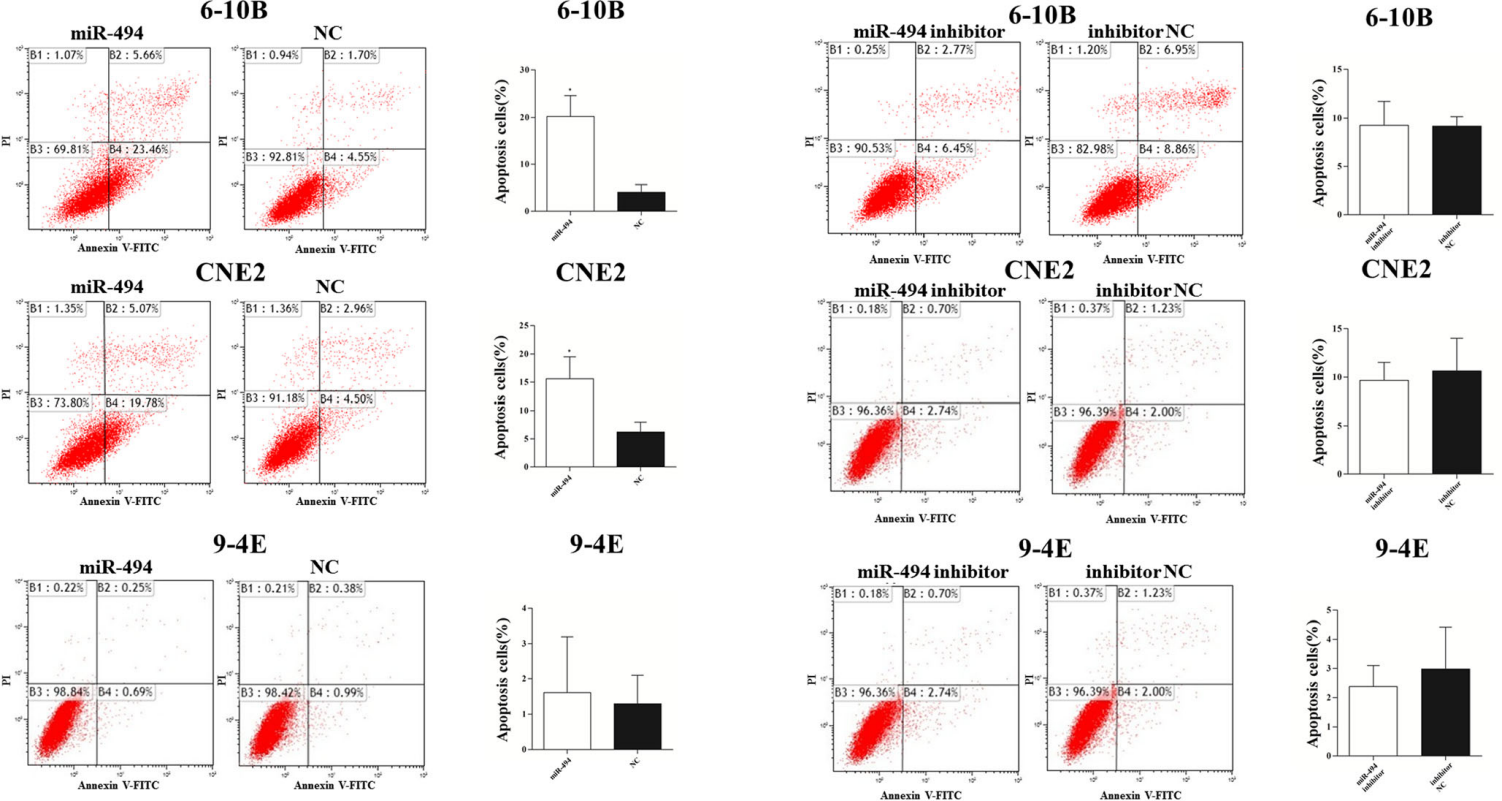
Effects of miR-494 on the apoptosis in NPC cells. The apoptosis rates were performed by the flow cytometry analysis after 6-10B, 9-4E, and CNE2 cells were transfected with miR-494 mimic, negative control, miR-494 inhibitor, or inhibitor negative control. Data are presented as mean ± SD (*p < 0.05, **p < 0.001)

To investigate the effect of miR-494 on the apoptosis of NPC cells, flow cytometry was performed to detect the apoptosis rate. Compared with the cells transfected with negative control, the cell apoptosis rate was restrained significantly in cells transfected with miR-494 mimic, with 20.2 vs 4.1 % in 6-10B cells (*p* = 0.004), and 15.6 vs 6.2 % in CNE2 cells (*p* = 0.019). However, it was not so obvious in 9-4E cells, with the apoptosis rate being 1.6 vs 1.3 % (*p* = 0.783). We did not observe significant change of apoptosis when the cells were transfected with miR-494 inhibitor. The cell apoptosis rate was 9.3 vs 9.2 % (*p* = 0.956), 2.4 vs 3.0 % (*p* = 0.554), and 9.7 vs 10.6 % (*p* = 0.686) in 6-10B, 9-4E, and CNE2 cells, respectively, when the cells were transfected with miR-494 inhibitor and inhibitor negative control (Fig. 5).

### GALNT7 and CDK16 are the potential targets of miR-494

**Fig. 6:**
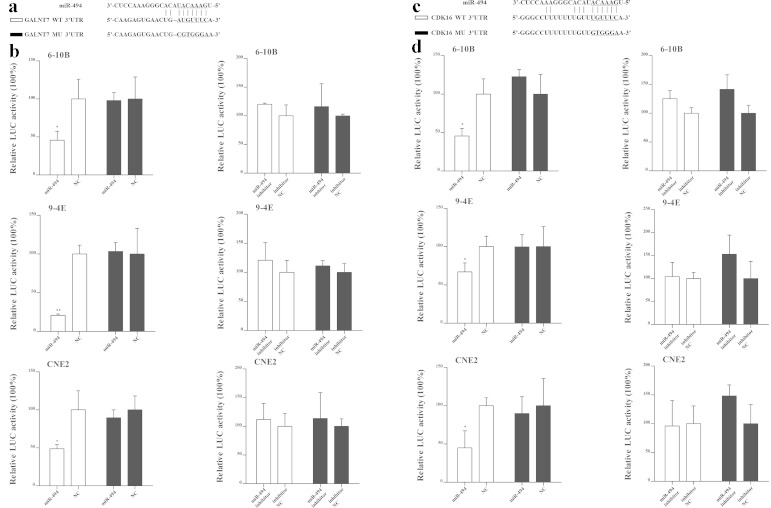
GALNT7 and CDK16 are two target genes of miR-494. **a**, **c** Four fragments of GALNT7 and CDK16 3′-UTR were constructed, which contained the wild-type (WT) potential binding sites of miR-494 and the mutated sequence (MU). **b**, **d** The psiCHECK^TM^-2 luciferase constructs containing WT or MU sequence were transfected together with miR-494 mimic, negative control, inhibitor, or inhibitor negative control into 6-10B, 9-4E, and CNE2 cells. Luciferase activity was measured through the dual luciferase assay system. Data are presented as mean ± SD (*p < 0.05, **p < 0.001)

Bioinformatic analysis (miRWalk) was used to determine the potential targets of miR-494, and two oncogenes, GALNT7 and CDK16, were selected as potential targets for further study (Fig. 6a, c). The luciferase reporter assay validated that the activity of the reporter containing 3′-UTR of GALNT7 was decreased when the cells were transfected with miR-494 mimic in 6-10B cells (*p* = 0.030), 9-4E cells (*p* < 0.001), and CNE2 cells (*p* = 0.026) (Fig. 6b), while that of CDK16 was also decreased in three NPC cells (*p* = 0.013, *p* = 0.034, and *p* = 0.018, respectively) (Fig. 6d). However, the activity of these reporters did not change when the three types of NPC cells were transfected with miR-494 inhibitor. For further verification of the binding sites of miR-494, the seed sequences on the 3′-URT of GALNT7 and CDK16 were mutated (Fig. 6a, c). The activity of the reporter containing the mutated seed sequence had no obvious change when the cells were transfected with either miR-494 mimic or miR-494 inhibitor (Fig. 6b, d).

### MiR-494 down-regulated the protein levels of GALNT7 and CDK16 in NPC

**Fig. 7:**
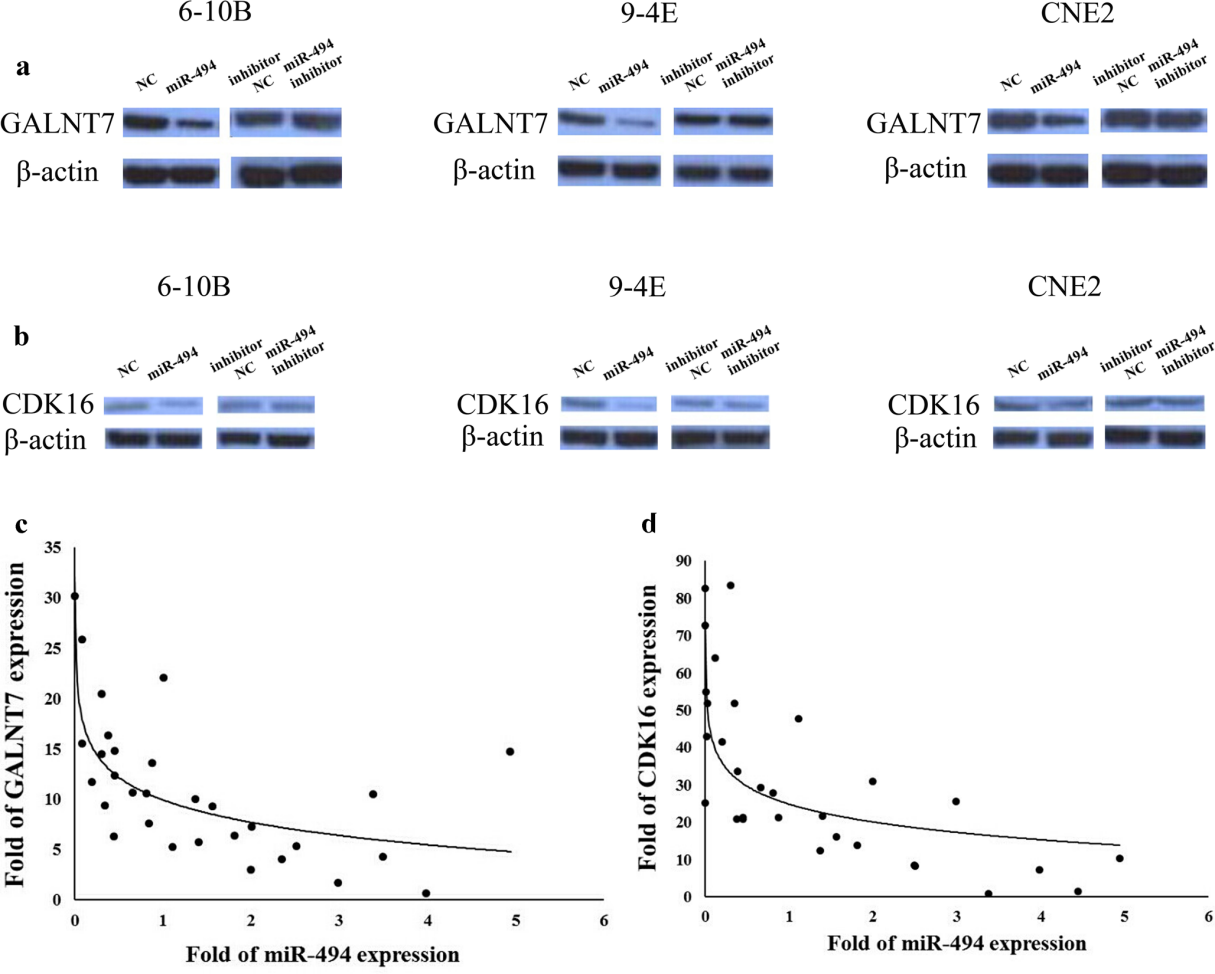
MiR-494 down-regulated the expression of GALNT7 and CDK16. **a**, **b** Western blotting showed that the function of miR-494 on GALNT7 and CDK16 expression at protein level in 6-10B, 9-4E, or CNE2 cells after transfection of miR-494 mimic, negative control, inhibitor, or inhibitor negative control. Date are presented as mean ± SD (*p < 0.05, **p < 0.001). **c**, **d** The mRNA expression level of GALNT7 and CDK16 negatively correlated with miR-494 in 30 NPC tumor samples by qRT-PCR. 2^−ΔΔCT^ method was used to analyze the data

The protein expression levels of GALNT7 or CDK16 were examined by Western blotting in 6-10B, 9-4E, and CNE2 cells. MiR-494 mimic significantly down-regulated the protein levels of GALNT7 and CDK16. However, miR-494 inhibitor did not show obvious effect on the levels of GALNT7 and CDK6 (Fig. 7a, b).

The expression of miR-494, GALNT7, and CDK16 was examined in 30 freshly frozen NPC clinical specimens and 20 normal nasopharyngeal epithelial tissues. Their expression pattern showed that the levels of GALNT7 or CDK16 decreased when miR-494 expression increased, suggesting that GALNT7 and CDK16 were indeed regulated by miR-494 in tissue samples (Fig. 7c, d).

## Discussion

MiRNAs play important roles in a variety of cancers in which these miRNAs were up-regulated or down-regulated and functioned as either oncogenes or tumor suppressors [22, 23]. We screened for differentially expressed miRNAs in NPC and validated that miR-494 was dramatically down-regulated in NPC tissues and NPC-derived cells. The low expression of miR-494 in different cancers suggests that it might have anticancer effects. In the present study, we extensively investigated the role of miR-494 and indentified its targets in NPC cells.

Previous studies reported that miR-494 influenced the occurrence and development of tumor. For instance, miR-494 was involved in the proliferation, senescence, and apoptosis of lung cancer by targeting downstream genes [18, 19]. Shen PF [24] identified that miR-494 was down-regulated in prostate cancer and significantly inhibited the proliferation, metastasis, and invasion of prostate cancer cells by regulating CXCR4 which involved in many fundamental cellular process and cancer progression. Accordingly, our study revealed that over-expression of mir-494 could suppress the proliferation, migration, and invasion of NPC cells and promote apoptosis dramatically. However, in 9-4E cells, the effect of miR-494 to promote apoptosis was not so obvious as that in 6-10B and CNE2 cells, suggesting that the intrinsic characteristics of certain cell types may affect their response to miR-494.

GALNT7 is a member of the GalNAc-transferase family which encodes GalNAc-transferase 7. GALNT7 is very important as a follow-up enzyme in the first step of O-glycosylation. O-glycans’ alteration leads to certain biological behavior by changing the connection between cancer cells and the environment around them. The changes in several cancer-associated structures of the O-glycans can alter the characteristics participating in cancer invasion and metastasis and other functions of cells [25]. GalNActransferases initiates mucin-type O-linked glycosylation in the Golgi apparatus by impelling the transfer of GalNAc to serine and threonine residues on targeting proteins to affect the biological consequences of the cell [26]. At the same time, GALNT7 is reported to be up-regulated in many cancers such as laryngeal carcinoma, cervical cancer, and pancreatic cancer [27–29]. Recently, it is reported that suppressing the expression of GALNT7 can inhibit cell invasion and metastasis [30]. Meanwhile, another target gene, CDK16, which belongs to the CDK2 subfamily, may take part in cell cycle regulation and play a critical role during G_1_ to S phase transition. Thus, CDK2 subfamily is frequently studied on the effect of cell proliferation and differentiation [31, 32]. CDK16 might be a part of cellular protein networks and multiple signal transduction cascades by interacting with other four intracellular proteins [33]. As is known, lots of cell functions depend on signaling from the cytoskeleton to the nucleus. CDK16 could start a kinase cascade by connecting with CDK5 to govern cytoskeletal rearrangements essential for neuron migration and neurite outgrowth [34]. In our study, we validated that GALNT7 and CDK16 are direct targets of miR-494. MiR-494 could inhibit the protein expression of GALNT7 or CDK16, and the expression levels of miR-494 and its targets were trans-correlated in NPC tissues.

As we have shown, the expression level of miR-494 was prominently down-regulated in three NPC-derived cells, so further inhibition of miR-494 may not result in more observable influence on the occurrence and development in NPC, such as the NPC-derived cells apoptosis and the target genes interference effect. On the other hand, miR-494 belongs to the miR-154 family which has been found containing 18 members locating in 14q32.31 (miRBase). The members are verified as oncogenes or tumor suppressors in some tumors such as liver cancers, gliomas, prostate cancer, and gastric cancer [35–38]. Therefore, we thought that the other members of miR-154 family could play similar roles in regulating target genes by binding to the different potential sites and further inhibition of miR-494 may not result in obvious functional changes. Whether the other members of miR-154 family play prominent roles in NPC and the precise function of GALNT7 and CDK16 in NPC still need to be further verified in our future study.

Overall, our study provided evidence showing that miR-494 might be an important regulator for NPC tumorigenesis.
